# Supplementary material for: Insights into magma ocean dynamics from the transport properties of basaltic melt
Source: Nat Commun. 2022 Dec 8;13:7590. doi: 10.1038/s41467-022-35171-y (PMC9731987; doi:10.1038/s41467-022-35171-y)
Supplement: Supplementary file 1 — Supplementary Information [file 41467_2022_35171_MOESM1_ESM.pdf]

Supplementary Information for

Insights into magma ocean dynamics from the transport properties of basaltic melt

Suraj K Bajgain[1,2], Aaron Wolfgang Ashley[1], Mainak Mookherjee[1#], Dipta B Ghosh[3], Bijaya B Karki[3~]*

[1]Earth Materials Laboratory, Earth, Ocean and Atmospheric Sciences, Florida State University, Tallahassee, FL 32306, USA

[2]Department of Geology, School of Natural Resources & Environment, Lake Superior State University, Sault Ste. Marie, MI 49783, USA

[3]School of Electrical Engineering and Computer Science, Department of Geology and Geophysics, Center for Computation and Technology, Louisiana State University, Baton Rouge, LA 70803, USA

*sbajgain@lssu.edu, #mmookherjee@fsu.edu, ~bbkarki@lsu.edu

Supplementary Note 1

The atomic coordination, which is often used to characterize the local structure, can be calculated for a given species α with respect to another species β from

$$C_{\alpha\beta} = 4\pi\rho_\beta \int_0^{r_{min}} r^2 g_{\alpha\beta}(r)\, dr \qquad (1)$$

where ρ_β is the number density of species β. $g_{\alpha\beta}$ is the partial RDF for the atom pair α and β is defined as-

$$g_{\alpha\beta}(r) = \frac{1}{4\pi\rho_\beta r^2}\left[\frac{dN_\beta(r)}{dr}\right] \qquad (2)$$

where, N_β is the number of species β, within a sphere of radius r around a selected atom of type α. This nearest neighbor coordination is the number of atoms of species β, which are within a distance from an atom of species α within a radius defined by the r_{min}, i.e., a minimum radial distance after the first peak in the RDF of atomic pair α and β.

Partial radial distribution functions (PRDF) of all cation-oxygen pairs exhibit short-range order and long-range disorder which are characteristics of the liquid state (**Figure S1**). Our results show that Si-O bond length initially increases with pressure up to ~30 GPa and is roughly

constant at greater pressures (**Figure S2**). Average Al-O bond length shows similar behavior, but Al-O bond length decreases with compression > 20 GPa (**Figure S2**). The average bond lengths of both Al-O and Si-O coordination pairs decrease with decreasing temperature. However, the effect of temperature on average coordination numbers appears negligible. We find that average cation-oxygen coordination numbers for all species show prompt increases with compression below 30 GPa, whereas at pressures > 30 GPa the average coordination increases more gradually (**Figure S2**). Both the average Al-O and Si-O coordination numbers are ~4 at ambient pressure, i.e., nearly all Al and Si cations are in 4-fold coordination with oxygen. As pressure increases to ~30 GPa, the average Si-O coordination increases from ~4 to ~5 with only about 25 % of silicon atoms in 4-fold coordination with oxygen; the abundance of SiO_5 and SiO_6 at these conditions are ~50% and ~25%, respectively. When the basaltic melt is compressed to core-mantle boundary (CMB) pressures, the average Si-O coordination transitions to 6-fold coordination. The four-fold to the six-fold transformation of Si-O coordination has also been observed in previous studies for wide range of silicate melt compositions[1-4]. At ambient pressures, basaltic melts contain 48 % non-bridging oxygen atoms (NBO) at 4000 K, 41 % NBOs at 3000 K, and 39 % NBOs at 2200 K (**Figure S3**). The NBOs continuously decreases with increasing pressure along all isotherms. The NBO species are replaced by polymerized bridging oxygen atoms (BO) at lower pressures and oxygen tricultures (OT_3) at high pressures (**Figure S3**).

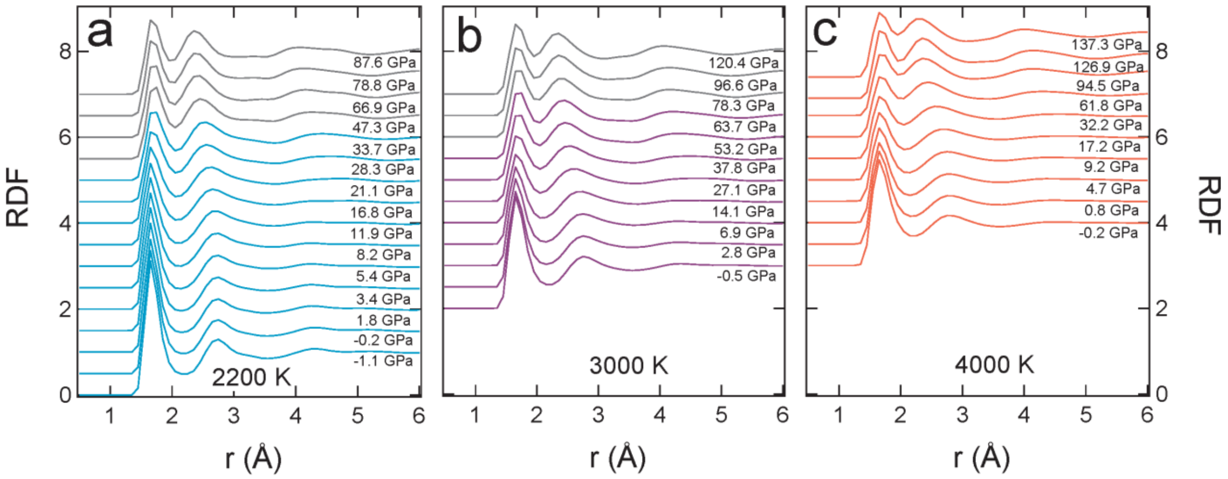

Figure S1 Total radial distribution function (RDF) at **(a)** 2200 K, **(b)** 3000 K, and **(c)** 4000 K. Grey lines in panel (a) and (b) shows the pressure points that are not used in the discussion of results.

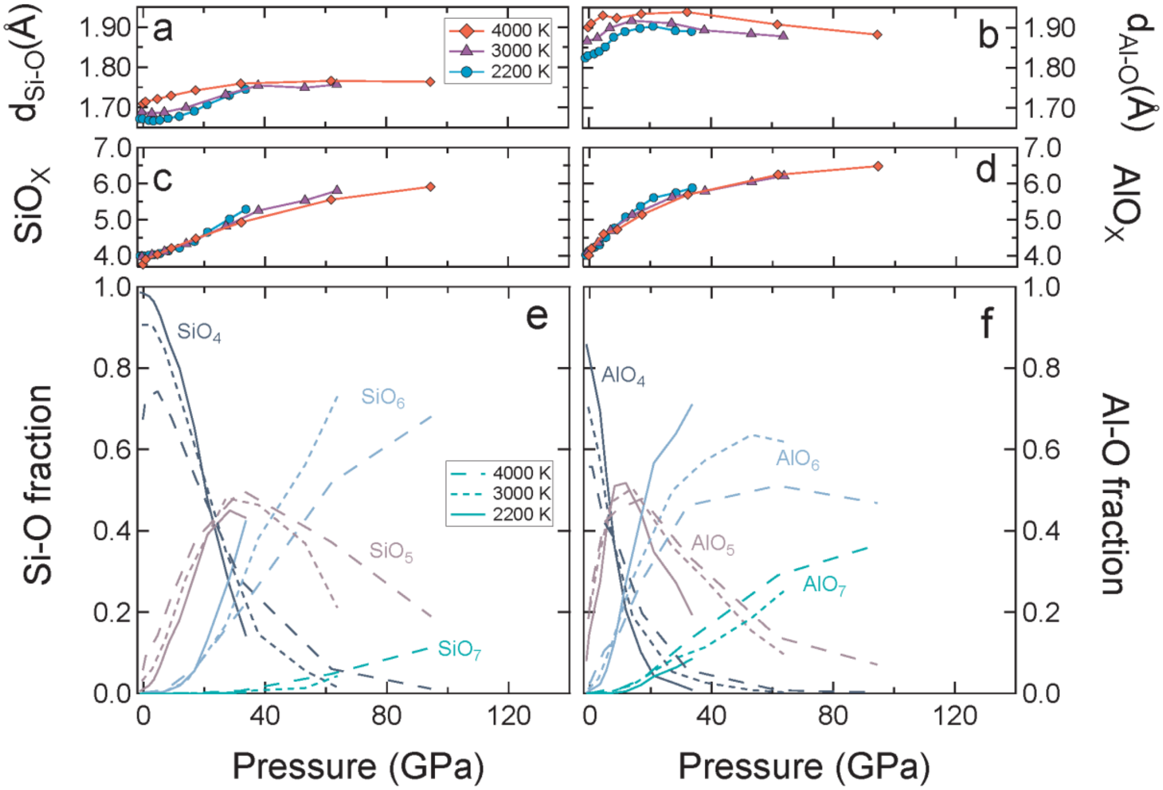

Figure S2 Average bond distances as a function of pressure for Al and Si cations with oxygen: **(a)** Si-O and **(b)** Al-O at 2200 K (cyan circles with line), 3000 K (purple triangles with line), and 4000 K (red diamonds with lines). Average **(c)** Si-O and **(d)** Al-O coordination numbers along all isotherms. Fraction of **(e)** Si-O and **(f)** Al-O coordination environments at 2200 K (solid lines), 3000 K (dashed lines), and 4000 K (long dashed lines).

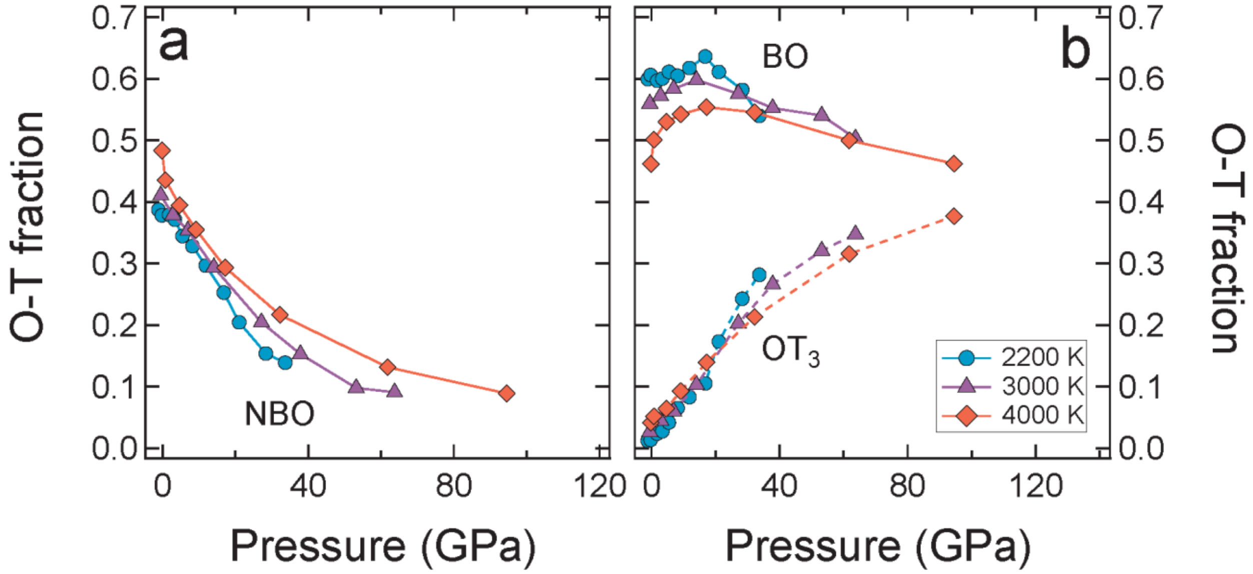

Figure S3 (a) Fraction of non-bridging oxygens (NBO) as a function of pressure at 2200 K (cyan circles with line), 3000 K (purple triangles with line), and 4000 K (red diamonds with lines). **(b) The proportion** of bridging oxygens, BO (one oxygen shared with 2 T's where T refers to cations Al and/or Si), and oxygen triclusters, OT_3 (oxygen shared by 3 T's).

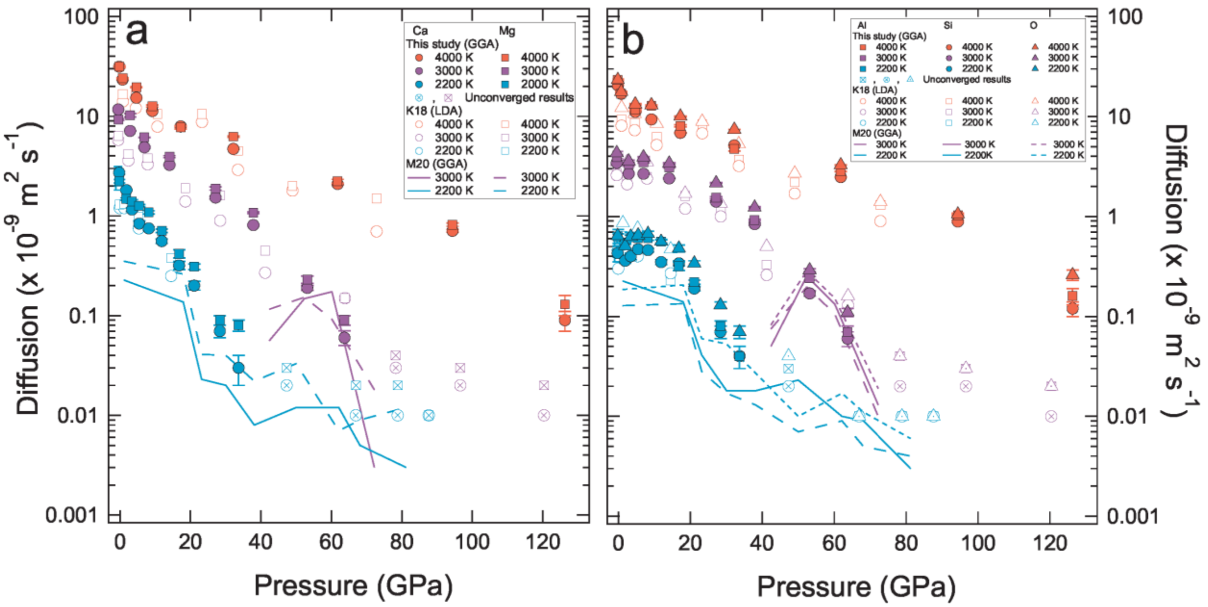

Figure S4 Diffusivities of (**a**) Ca, Mg and (**b**) Al, Si, O in basaltic melts along 2200, 3000 K, and 4000 K isotherms. Open symbols with crosses show the results from simulations that did not reach adequate convergence during our simulation timescales. Open symbols in both figures represent the results for previous *first-principles* molecular dynamics (FPMD) simulation using the local density approximation (LDA) methods[5]. Lines are the results from recent FPMD simulations[6].

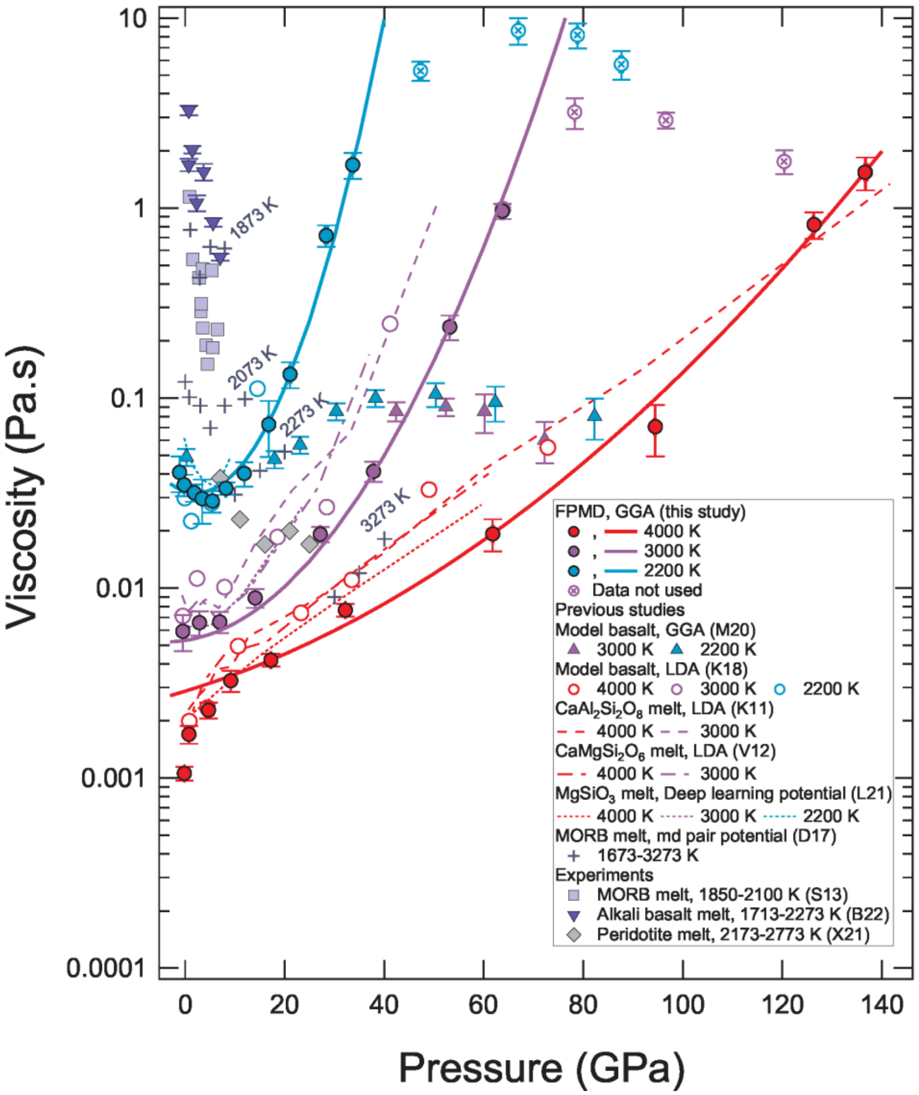

Figure S5 Viscosity of model basalt melt along different isotherms: 4000 K (filled red circles), 3000 K (filled purple circles), and 2200 K (filled cyan circles) as a function of pressure (this study, GGA). Viscosity can be well described using a modified Vogel-Fulcher-Tammann (VFT) equation. Filled triangles show the results from recent previous GGA calculations at 3000 K (purple) and 2200 K (cyan)[6]. Open circles represent previous first-principles results using location density approximation (LDA)[5]. Purple-filled squares, filled downward-facing triangles and yellow-filled diamonds are the experimental viscosity data for multi-component silicate melts: MORB (S13)[7], alkali basalt (B22)[8], and peridotite (X21)[9], respectively. Plus symbols denote the viscosity of MORB melt from previous pair potential molecular dynamics simulation[10]. The dashed curves indicate the viscosities of endmember composition diopside[11] and anorthite[12] melts along 4000 K and 3000 K from the previous FPMD studies that used LDA pseudopotentials. The dotted lines are the viscosity of $MgSiO_3$ melt calculated using deep learning potentials[13]. Circles with crosses are the viscosity results that are not statistically converged.

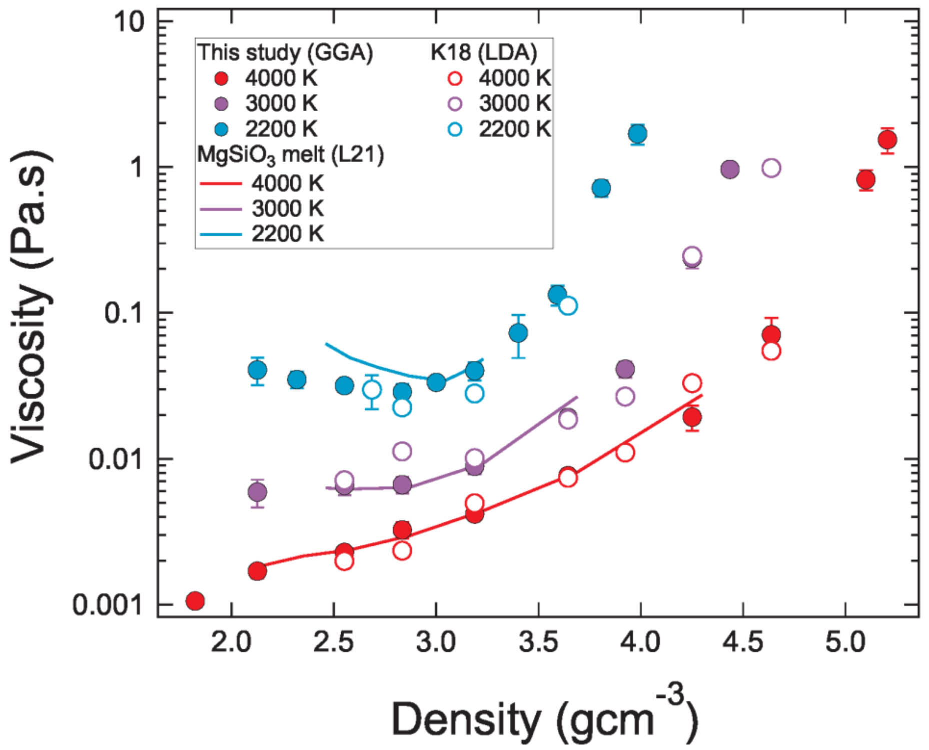

Figure S6 Viscosity of basaltic melt as a function of density. Solid circles represent the viscosity results from this study at 4000 K (red), 3000 K (purple), and 2200 K (cyan). Open symbols show the viscosity of model basalt from previous computational FPMD simulations studies which used LDA-based energy exchange correlation functionals[5]. Lines represent the viscosity of $MgSiO_3$ melt from a recent computational study using deep learning potential[13].

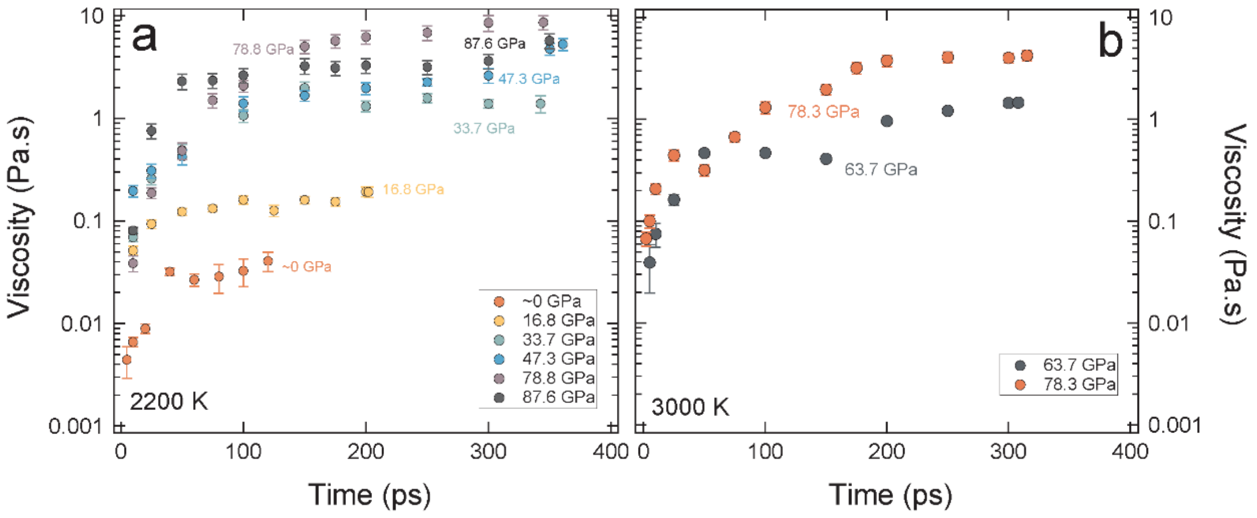

Figure S7 Viscosities at selected pressures calculated using different timescales at (**a**) 2200 K, and (**b**) 3000 K. The plot shows that viscosity asymptotically increases with increasing simulation time. Viscosity measurements collected before the asymptote are likely not representative of the equilibrated or statistically converged melt viscosity.

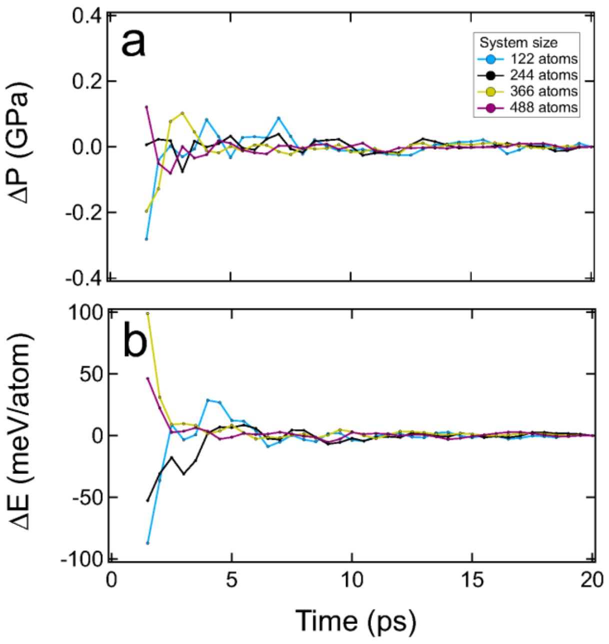

Figure S8 Differences of the **(a)** pressure and **(b)** energy averages taken over a period ranging between when the simulation began and a subsequent instant of time during the simulation run. The results are for ambient pressure and 6000 K. The blue, black, yellow, and pink lines are the results for basaltic melt with different simulation cell sizes with 122, 244, 366, and 488 atoms, respectively.

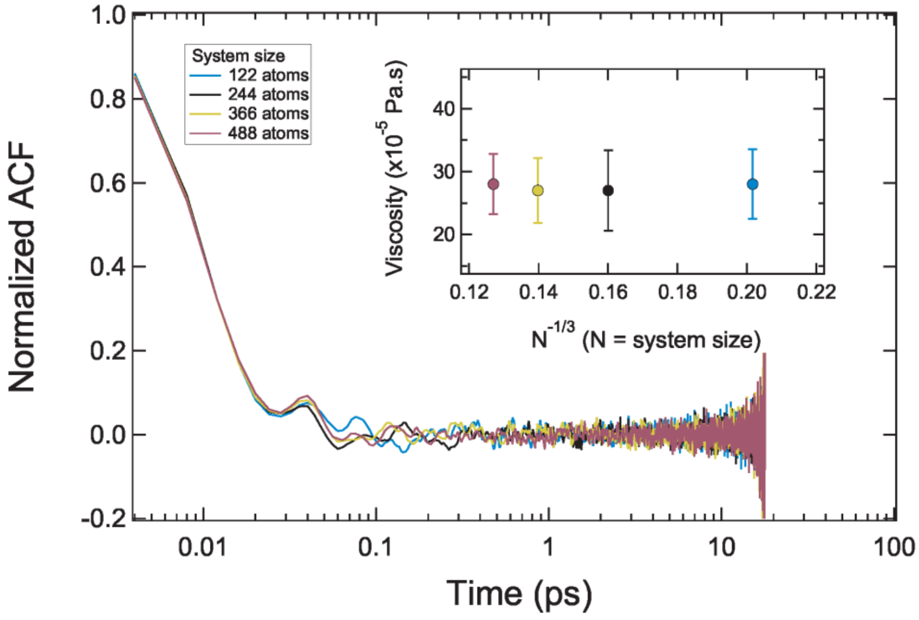

Figure S9 Normalized stress autocorrelation function (ACF) for viscosity calculations at ~0 GPa and 6000 K. The inset shows the corresponding viscosities plotted as a function of $N^{-1/3}$ where N is the total number of atoms in the simulation cell. The blue, black, yellow, and pink colors represent results from the simulation box containing 122, 244, 366, and 488 atoms, respectively.

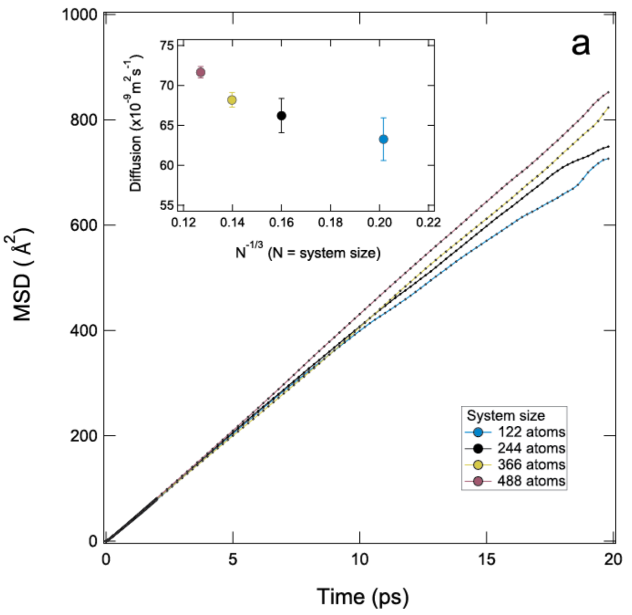

Figure 10 Plot of mean square displacement (MSD) as a function of simulation time at ~0 GPa and 6000 K. The inset shows the corresponding diffusion plotted as a function of $N^{-1/3}$ where N is the total number of atoms in the simulation cell. The blue, black, yellow, and pink colors represent results from the simulation box containing 122, 244, 366, and 488 atoms, respectively.

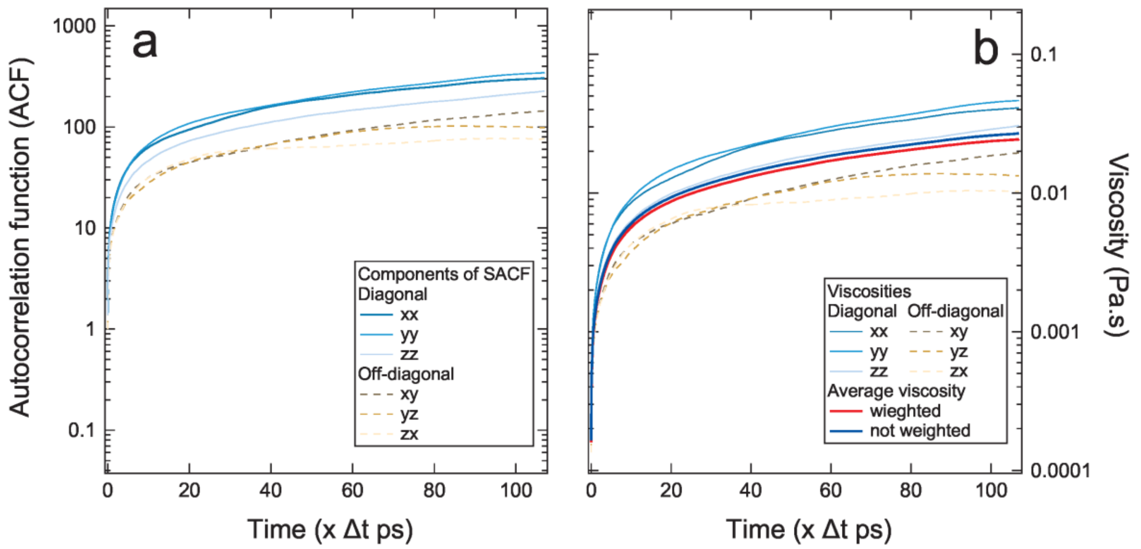

Figure 11 (a) Diagonal (solid lines) and off-diagonal (dashed lines) component of stress auto-correlation function (SACF) at ambient pressure and 2200 K (Δt=10) as a function of simulation time. **(b)** Viscosity is calculated along each diagonal (solid lines) and off-diagonal (dashed lines) component of the stress tensor. The thick blue line shows the simple average with the viscosity of each tensor weighted equally. The Red line shows the melt viscosity calculated using both the off-diagonal and diagonal components of the stress tensor with weighting factors of 1 and 4/3, respectively.

Supplementary References

1 Stixrude, L. & Karki, B. Structure and freezing of $MgSiO_3$ liquid in Earth's lower mantle. *Science* **310**, 297-299 (2005).

2 Solomatova, N. V., Caracas, R. & Manning, C. E. Carbon sequestration during core formation implied by complex carbon polymerization. *Nature Communications* **10**, 789 (2019).

3 Bajgain, S., Ghosh, D. B. & Karki, B. B. Structure and density of basaltic melts at mantle conditions from first-principles simulations. *Nature Communications* **6**, 9578, doi:10.1038/ncomms9578 (2015).

4 Bajgain, S. K., Ghosh, D. B. & Karki, B. B. First-principles simulations of CaO and CaSiO3 liquids: structure, thermodynamics and diffusion. *Physics and Chemistry of Minerals*, 1-12 (2015).

5 Karki, B. B., Ghosh, D. B. & Bajgain, S. K. in *Magmas Under Pressure* 419-453 (Elsevier, 2018).

6 Majumdar, A., Wu, M., Pan, Y., Iitaka, T. & John, S. T. Structural dynamics of basaltic melt at mantle conditions with implications for magma oceans and superplumes. *Nature communications* **11**, 1-9 (2020).

7 Sakamaki, T. *et al.* Ponded melt at the boundary between the lithosphere and asthenosphere. *Nat. Geosci.* **6**, 1041-1044 (2013).

8 Bonechi, B. *et al.* Experimental measurements of the viscosity and melt structure of alkali basalts at high pressure and temperature. *Scientific Reports* **12**, 1-12 (2022).

9 Xie, L. *et al.* Direct Viscosity Measurement of Peridotite Melt to Lower-Mantle Conditions: A Further Support for a Fractional Magma-Ocean Solidification at the Top of the Lower Mantle. *Geophysical Research Letters* **48**, e2021GL094507 (2021).

10 Dufils, T., Folliet, N., Mantisi, B., Sator, N. & Guillot, B. Properties of magmatic liquids by molecular dynamics simulation: The example of a MORB melt. *Chem. Geol.* **461**, 34-46 (2017).

11 Verma, A. K. & Karki, B. B. First-principles study of self-diffusion and viscous flow in diopside ($CaMgSi_2O_6$) liquid. *American Mineralogist* **97**, 2049-2055 (2012).

12 Karki, B. B., Bohara, B. & Stixrude, L. First-principles study of diffusion and viscosity of anorthite ($CaAl_2Si_2O_8$) liquid at high pressure. *American Mineralogist* **96**, 744-751 (2011).

13 Luo, H., Karki, B. B., Ghosh, D. B. & Bao, H. Anomalous Behavior of Viscosity and Electrical Conductivity of $MgSiO_3$ melt at Mantle Conditions. *Geophysical Research Letters* **48**, e2021GL093573 (2021).
